# Identification of the ataxin-1 interaction network and its impact on spinocerebellar ataxia type 1

**DOI:** 10.1186/s40246-022-00404-0

**Published:** 2022-07-29

**Authors:** Jiu-Ming Chen, Shi-Kai Chen, Pei-Pei Jin, Shun-Chang Sun

**Affiliations:** Department of Laboratory Medicine, Ruijin Hospital, Shanghai Jiao Tong University School of Medicine, Shanghai, 201801 China

**Keywords:** Spinocerebellar ataxia type 1 (SCA1), Ataxin-1, Protein–protein interaction, Polyglutamine expansion, Pathway analysis

## Abstract

**Background:**

Spinocerebellar ataxia type 1 (SCA1) is a neurodegenerative disease caused by a polyglutamine expansion in the ataxin-1 protein. The pathogenic mechanism resulting in SCA1 is still unclear. Protein–protein interactions affect the function and stability of ataxin-1.

**Methods:**

Wild-type and mutant ataxin-1 were expressed in HEK-293T cells. The levels of expression were assessed using real-time polymerase chain reaction (PCR) and Western blots. Co-immunoprecipitation was done in HEK-293T cells expressing exogenous wild-type and mutant ataxin-1 using anti-Flag antibody following by tandem affinity purification in order to study protein–protein interactions. The candidate interacting proteins were validated by immunoprecipitation. Chromatin immunoprecipitation and high-throughput sequencing and RNA immunoprecipitation and high-throughput sequencing were performed using HEK-293T cells expressing wild-type or mutant ataxin-1.

**Results:**

In this study using HEK-293T cells, we found that wild-type ataxin-1 interacted with MCM2, GNAS, and TMEM206, while mutant ataxin-1 lost its interaction with MCM2, GNAS, and TMEM206. Two ataxin-1 binding targets containing the core GGAG or AAAT were identified in HEK-293T cells using ChIP-seq. Gene Ontology analysis of the top ataxin-1 binding genes identified *SLC6A15*, *NTF3*, *KCNC3*, and *DNAJC6* as functional genes in neurons in vitro. Ataxin-1 also was identified as an RNA-binding protein in HEK-293T cells using RIP-seq, but the polyglutamine expansion in the ataxin-1 had no direct effects on the RNA-binding activity of ataxin-1.

**Conclusions:**

An expanded polyglutamine tract in ataxin-1 might interfere with protein–protein or protein–DNA interactions but had little effect on protein–RNA interactions. This study suggested that the dysfunction of protein–protein or protein–DNA interactions is involved in the pathogenesis of SCA1.

## Introduction

Spinocerebellar ataxia type 1 (SCA1) is an autosomal dominant neurodegenerative disease caused by a polyglutamine expansion in ataxin-1 [1]. The *ATXN1* gene was mapped to 6p22.3 and the mutation was identified as an unstable CAG expansion leading to altered protein function [2]. The detailed pathogenic mechanism resulting in SCA1 has not been determined. However, some studies suggest a complex pathogenic mechanism with contributions of both gain-of-toxic function from mutant ataxin-1 and loss of function from normal ataxin-1 [3, 4]. The normal function of ataxin-1 is still unclear. It is essential to decipher the pathogenic mechanism in SCA1 to understand the normal function of ataxin-1 in cellular processes. The study of protein–protein interactions holds the promise of revealing new protein relationships. Interacting protein partners might contribute to ataxin-1 function and stability [5]. The known ataxin-1-interacting protein partners include the 14-3-3 protein, transcriptional regulator Capicua (CIC), RNA-binding motif protein 17 (RBM17), the splicing factor U2AF 65 kDa subunit (U2AF65) [6–8]. Aberrant protein interactions and novel complexes formed by misfolded proteins are thought to mediate the neurotoxicity in polyglutamine diseases [9].

Nevertheless, it is unknown whether the neurotoxicity of aberrant proteins occurs through associations with native proteins, loss of associations, or atypical interactions in which misfolded proteins form new complexes. A deeper understanding of ataxin-1 binding partners will initiate new studies to explore SCA1 disease pathogenesis. Loss of function might contribute to neuronal dysfunction through abnormal protein interactions [4]. Tandem affinity purification (TAP) tagging was developed to study protein–protein interactions under intrinsic conditions of the cell using high-throughput techniques. In this study, we observed that in HEK-293T cells using TAP tagging, ataxin-1 interacted with mini-chromosome maintenance complex component 2 (MCM2), guanine nucleotide-binding protein G subunit alpha isoforms short (GNAS), and transmembrane protein 206 (TMEM206). Mutant ataxin-1 could result in the loss of normal protein–protein interactions in vitro.

There is evidence that ataxin-1 is involved in transcriptional regulation through binding to DNA and RNA in the nucleus [7, 10]. To elucidate the mechanisms of the transcriptional regulation of ataxin-1, we used chromatin immunoprecipitation high-throughput sequencing (ChIP-seq) and RNA immunoprecipitation high-throughput sequencing (RIP-seq) techniques to identify direct transcriptional targets of ataxin-1 in HEK-293T cells that expressed wild-type or mutant ataxin-1. Two ataxin-1 binding targets containing the core GGAG or AAAT were identified in HEK-293T cells using ChIP-seq in this study. A Kyoto Encyclopedia of Genes and Genomes (KEGG) pathway analysis of ataxin-1 binding targets revealed that the most significant network was axon guidance, which participates in adult brain repair and regeneration after brain injury. We identified thousands of RNAs that specifically associated with ataxin-1 using RIP-seq (https://www.ncbi.nlm.nih.gov/Traces/study/?acc=PRJNA688985). A Gene Ontology (GO) classification analysis of ataxin-1 binding targets revealed that the most significant networks were cellular nitrogen compound metabolic processes and biosynthetic processes. These results revealed a complex mechanism with contributions from the toxic gain-of-function of the mutant ataxin-1 and the loss of function of the wild-type ataxin-1. More studies are needed to explore the ataxin-1 function.

## Methods

### Protein expression

Wild-type *ATXN1* (30 CAG-repeat) was isolated from an RNA library derived from a human cerebellum (Clontech) by PCR cloning. A pair of primers (forward: 5'-TTCGAATTCTGatgaaatccaaccaagagc-3'; reverse: 5'-ACTTCTAGActacttgcctacattagacc-3') corresponding to the 5' and 3' ends of the *ATXN1* gene were inserted into the expression vector pLVX-Puro-3xFlag-SBPtag2 (FitGene) and digested with *Eco*RI and *Xba*I (Thermo Scientific). Mutant *ATXN1* (61 CAG-repeat) cDNA was synthetized chemically and inserted into the pLVX-Puro-3xFlag-SBPtag2 expression vector and then digested with *Eco*RI and *Xba*I. The plasmid, pLVX-Puro-3xFlag-SBPtag2-Atx1-W encoding wild-type *ATXN1*, and the plasmid, pLVX-Puro-3xFlag-SBPtag2-Atx1-M encoding mutant *ATXN1*, were constructed by PCR cloning using Pfu polymerase (Promega). Both wild-type and mutant *ATXN1* constructs were sequenced prior to transfection to exclude undesired mutations.

HEK-293T cells were plated at a density of 5 × 10^5^ cells in six-well plates, and co-transfected with 2.0 µg of the plasmids, pLVX-Puro-3xFlag-SBPtag2-Atx1-W/ pLVX-Puro-3xFlag-SBPtag2-Atx1-M, 1.0 µg pMD2.G envelope vector (Invitrogen), and 1.5 µg psPAX2 packaging vector (Invitrogen) by the PolyFect^®^ transfection reagent (Qiagen), according to the manufacturer’s recommendations. The medium was changed 24 h after transfection, and 2 μg/ml puromycin was added to the plates 72 h after transfection for selection. Subsequently, the cells were exposed to 2 μg/ml puromycin for 14 days. Puromycin-resistant cells were taken and cultured in separate flasks containing medium supplemented with 2 μg/ml puromycin. The resistant cells were considered to be stably transfected cell lines. The HEK-293T cell line, which was left untreated, served as the negative control.

Gene expression was assessed using real-time polymerase chain reaction (PCR) and Western blots. Total RNA was extracted from lysates prepared from cultured HEK-293T cells using an RNeasy mini kit (Qiagen). RNA concentration and purity were determined using a microspectrophotometric method. Only pure RNA with A260/280 ratios greater than 1.8 was used for qPCR. cDNA was synthesized using a commercial Bestar qPCR RT Kit (DBI Bioscience) according to the manufacturer’s instructions. qPCR was performed in triplicate for each cDNA sample using an ABI 7500 Instrument (Life Technology) and 96-well microtiter plates. The expression levels of GAPDH were used as internal controls. The primer sequences used in the qPCR are shown in brackets (ATXN1-F: 5'-gatcgactccagcaccgtag-3'; ATXN1-R: 5'-gatgaccagccctgtccaaa-3'; GAPDH-F: 5'-tgcaccaccaactgcttagc-3'; GAPDH-R: 5'-ggcatggactgtggtcatgag-3'). Relative expression was calculated using the 2 − ^ΔΔCt^ method and normalized to the *GAPDH* gene.

Lysates from HEK-293T cells were extracted in lysis buffer (50 mM Tris–HCl, pH 7.4, 250 mM sucrose, 0.1% *β*-mercaptoethanol, 10 mM NaF, 1 mM sodium orthovanadate, 0.2 mM PMSF, 10 µg/ml Leupeptin, and 1% Triton, Sangon Biotech). Proteins were separated on 4–10% gradient SDS–polyacrylamide gels, then transferred onto polyvinylidene fluoride membranes (Amersham Bioscience). Protein transfer was confirmed by Coomassie brilliant blue G-250 staining. Membranes were blocked in Tris-buffered saline with 0.1% Tween^®^ 20 detergent (TBST) buffer containing 5% nonfat milk, and antibodies were diluted with the same solution. Blots were probed with an anti-Flag-HRP monoclonal antibody (Sigma-Aldrich, 1:1000 dilution). Goat anti-mouse secondary antibody linked to horseradish peroxidase at 1:3000 dilution (Sangon Biotech) was used for enhanced chemiluminescence immunodetection (Amersham Bioscience) according to the manufacturer’s recommendations. Band visualization for individual protein was generated by exposure of the blots to film.

### Co-immunoprecipitation

As described above, the stably transfected cell lines expressing human wild-type and mutant ataxin-1, and the HEK-293T cell line as a negative control were frozen at − 80 °C for 10 min after harvest, and then, cells were lysed. The lysates were centrifuged at 12,000 rpm for 15 min, and then, 50 µg aliquots of the supernatant were frozen as input samples. Pulldowns were done in HEK-293T cells transiently expressing exogenous wild-type and mutant ataxin-1. For immunoprecipitation, 500 μg of the cell lysate was incubated with 1 μg of anti-Flag M2 antibody-conjugated magnetic beads (Sigma-Aldrich) overnight at 4 °C. The following day, the antibody-conjugated magnetic beads were washed three times with lysis buffer, and immunocomplexes were eluted three times with sample buffer supplemented with 0.1% *β*-mercaptoethanol. The eluted immunocomplexes were prepared for Western blotting and liquid chromatography-mass spectrometer/mass spectrometer (LC−MS/MS). The eluted proteins were digested using 1 mg/ml trypsin (Gibco) and subjected to LC–MS/MS. Briefly, the peptides from trypsin digestion were separated using an EASY-nLC system (Proxeon Biosystems/ThermoFisher Scientific), according to the manufacturer’s instructions. The high-performance liquid chromatography (HPLC) system was coupled with a QExactive mass spectrometer by a nanoscale LC interface source (Proxeon Biosystems/ThermoFisher Scientific) equipped with a column oven (PRSO-V1, Sonation) set to 30 °C. Survey spectra (350−1800 m/z) were acquired with a resolution of 70,000 at 350 m/z and using an automatic gain control (AGC) to set an AGC target value of 3,000,000 charges. The 20 most intense ions from the high-resolution survey scan were sequenced by higher-energy collisional dissociation at a resolution of 17,500 and an AGC target value of 100,000 charges. Maximal filling times were set to ms for the MS/MS spectra. Precursor ion charge state screening was enabled, and unassigned charge states and singly charged species were rejected. The precursor ions were selected according to isotopic matching and laid on a dynamic exclusion list. The mass spectrometric data files acquired using the Xcalibur software (version 2.0) were analyzed using the MaxQuant suite of algorithms (version 1.3.0.5). The LC−MS/MS data were searched in the UniProt Knowledgebase human protein database (version Jul 4, 2020; 192, 656 entries). Enzyme specificity was based on trypsin, which allowed for cleavage at the N-terminal and proline position as well as between proline and aspartic acid. The minimum required peptide length was set to six amino acids. The required false discovery rate was set to 1% for the peptide and protein levels. The protein identification data from LC–MS were uploaded into Ingenuity. Pathway analysis was assigned to the selected candidates, ataxin-1-interacting proteins, and different biological processes were assessed based on evidence from the literature. The Ingenuity Pathway Analysis was performed to determine whether the candidate ataxin-1-interacting proteins could be mapped to neuronal functional networks.

Eluted proteins were assayed using SDS-PAGE. Briefly, the proteins were separated on 8% and 10% SDS-PAGE gels and transferred onto a polyvinylidene fluoride membranes (Amersham Bioscience). The membranes were blocked in 5% nonfat milk in TBST and incubated with anti-Flag antibody (Sigma-Aldrich, 1:1000 dilution) and anti-Myc antibody (Santa Cruz, 1:1000 dilution) in TBST for 1 h. After extensive washing with TBST, the membranes were incubated with horseradish peroxidase-coupled goat anti-mouse secondary antibody (Santa Cruz, 1:3000 dilution) diluted in TBST. The protein bands were visualized using an ECL enhanced luminescence detection kit (Sangon Biotech) according to the manufacturer’s instructions.

### Chromatin Immunoprecipitation and high-throughput sequencing

1 × 10^8^ HEK-293T cells expressing wild-type or mutant ataxin-1 were fixed with 1% formaldehyde for 10 min and then lysed and sonicated for 40 min in a Bioruptor sonicator (Diagenode) according to the manufacturer's instructions. The supernatants were pre-cleared using protein G agarose beads (GE Healthcare). Then, the chromatin samples were immunoprecipitated using an anti-Flag affinity-purified mouse polyclonal antibody (Sigma-Aldrich). Purified DNA samples were obtained from the precipitated chromatin and applied to the Illumina ChIP-Seq HT Sequencing Library construction platform using the ChIP-Seq Sample Prep Kit (Illumina), according to the manufacturer's instructions. Briefly, an approximately 300 bp fraction of DNA was isolated using agarose gel electrophoresis after adapter ligation. The clusters for sequencing were generated using the TruSeq PE Cluster Kit (Illumina). The DNA sequencing was conducted by Genewiz Bio Inc. (Suzhou, China) using HiSeq 2000 (Illumina) for one hundred bases in a single-read manner. The input DNA samples were sequenced as reference models for data analysis. The data were checked for quality through quantification using the comparative delta Ct method, such that the data reads were applied to the human genome alignment. All reads were aligned to the human genome (Hg19) using the BOWTIE program. Reads with more than three mismatches in the alignment were excluded.

### RNA immunoprecipitation and high-throughput sequencing

RNA immunoprecipitation was performed using 5 × 10^7^ HEK-293T cells expressing wild-type or mutant ataxin-1 following the instructions of the TruSeq RNA Sample Prep Kit (Illumina). Briefly, Flag-tagged wild-type and mutant ataxin-1 RNAs were precipitated from cell lysates via anti-Flag beads (Sigma-Aldrich). The RNA fraction was resuspended in QIAzol buffer (Qiagen). Negative control input samples were applied to the immunoprecipitation to identify the presence of any RNase contamination as part of the RNA quality assessment. All rRNAs were depleted using Illumina Ribo-Zero to ensure an appropriate depth of sequencing. RNA libraries for sequencing were constructed using RNA with good quality according to the manufacturer’s recommendations. Illumina high-throughput sequencing was carried out for the RNA libraries by Genewiz Bio Inc. (Suzhou, China). The end reads were mapped to the human genome (Hg19) using the BOWTIE aligner.

### Statistical analysis

The data were presented as means ± SD, and statistical analysis was conducted using GraphPad Prism 7 software. Student’s *t* test or one-way ANOVA (analysis of variance) was used to compare differences between groups. A value of *p* < 0.05 was defined as a statistically significant difference.

## Results

### Ataxin-1-interacting proteins

**Fig. 1:**
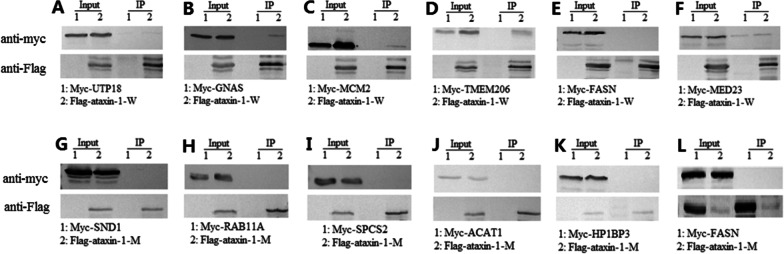
Validation of proteins associated with ataxin-1. **A**–**F** Co-immunoprecipitation experiments were performed with HEK-293T cells expressing wild-type ataxin-1. In each case, Flag was immunoprecipitated, and then Myc and Flag were immunoblotted. Co-immunoprecipitation-western assays showed that wild-type ataxin-1 interacts with GNAS (**B**), MCM2 (**C**), and TMEM206 (**D**), but could not interact with UTP18 (**A**), FASN (**E**), and MED23 (**F**). **G**–**L** Co-immunoprecipitation experiments were performed with HEK-293T cells expressing mutant ataxin-1. Similarly, Flag was immunoprecipitated, and then Myc and Flag were immunoblotted in each case. Co-immunoprecipitation-western assays demonstrated that mutant ataxin-1 could not interact with SND1 (**G**), RAB11A (**H**), SPCS2 (**I**), ACAT1 (**J**), HP1BP3 (**K**), and FASN (**L**). Molecular weight (kDa): Myc-UTP18 (62), Myc-GNAS (46), Myc-MCM2 (104), Myc-TMEM206 (40), Myc-FASN (273), Myc-MED23 (130), Myc-SND1 (104), Myc-RAB11A (25), Myc-SPCS2 (25), Myc-ACAT1 (46), Myc-HP1BP3 (63), Myc-FASN (273), Flag-ataxin-1-W (94), Flag-ataxin-1-M (120)

Western blots and qPCR revealed that wild-type and mutant ataxin-1 was expressed in HEK-293T cells. Both wild-type and mutant ataxin-1 proteins were isolated successfully from complexes using TAP. LC–MS analysis of the co-immunoprecipitated protein complexes identified and quantified more than 40 proteins using anti-Flag antibody following by TAP in the pooled wild-type and mutant ataxin-1 fractions. These results were based on three independent experiments after database searching and automatic validation using TPP tools. All identified proteins were present in a minimum of two of the three experiments. Non-transfected HEK-293T cells were grown in medium containing light Arg (L). HEK-293T cells expressing wild-type ataxin-1 were cultured in moderate Arg (M) medium, and HEK-293T cells expressing mutant ataxin-1 were cultured in heavy Arg (H) medium. Therefore, proteins with a relative abundance ratio (M/L or H/L) of greater than 1.2 were expected to be ataxin-1-interacting proteins. The candidate ataxin-1-interacting proteins were validated by immunoprecipitation using anti-Flag antibody for the proteins with the top six relative abundance ratios (M/L and H/L) for pooled wild-type and mutant ataxin-1 (see Table 1) fractions. Pulldowns were performed with HEK-293T cells transiently expressing exogenous wild-type or mutant ataxin-1 and candidate ataxin-1-interacting proteins. Co-immunoprecipitation analysis revealed the presence of ataxin-1 in Flag-immunoprecipitated complexes from cell extracts prepared from HEK-293T cells expressing wild-type ataxin-1 (Fig. 1B, C, and D), indicating that ataxin-1 was physically associated with GNAS, MCM2, and TMEM206 in HEK-293T cells. Our results demonstrated that no ataxin-1 was present in Flag-immunoprecipitated complexes from the cell extracts (Fig. 1A, E, and F). These data suggested that ataxin-1 interacted with GNAS, MCM2, and TMEM206 in HEK-293T cells. In the cell extracts prepared from HEK-293T cells transfected with mutant ataxin-1, ataxin-1 was not detected in Flag-immunoprecipitated complexes as seen in immunoblot images (Fig. 1G–L). Co-immunoprecipitation-Western blot assays could not exclude that mutant ataxin-1 did not interact with SND1, RAB11A, SPCS2, ACAT1, HP1BP3, and FASN due to the majority of mutant ataxin-1 remaining in a nuclear aggregated state.

**Table 1:** Summary of the top six M(H)/L ratio proteins identified by quantitative analysis of the TAP tagging in HEK-293T cells expressing wild-type and mutant ataxin-1

Candidate	Wild-type ataxin-1			Mutant ataxin-1		
	Accession	Protein	Ratio M/L	Accession	Protein	Ratio H/L
1	P49736	MCM2	4.457	Q5SSJ5	HP1BP3	10.442
2	Q9H813	TMEM206	2.545	P49327	FASN	1.896
3	Q9ULK4	MED23	2.056	P24752	ACAT1	1.771
4	P63092	GNAS	1.758	Q15005	SPCS2	1.498
5	Q9Y5J1	UTP18	1.705	P62491	RAB11A	1.342
6	P49327	FASN	1.607	Q7KZF4	SND1	1.256

The relative abundance ratios (M/L and H/L) > 1.2 were considered candidate ataxin-1-interacting proteins

**Fig. 2:**
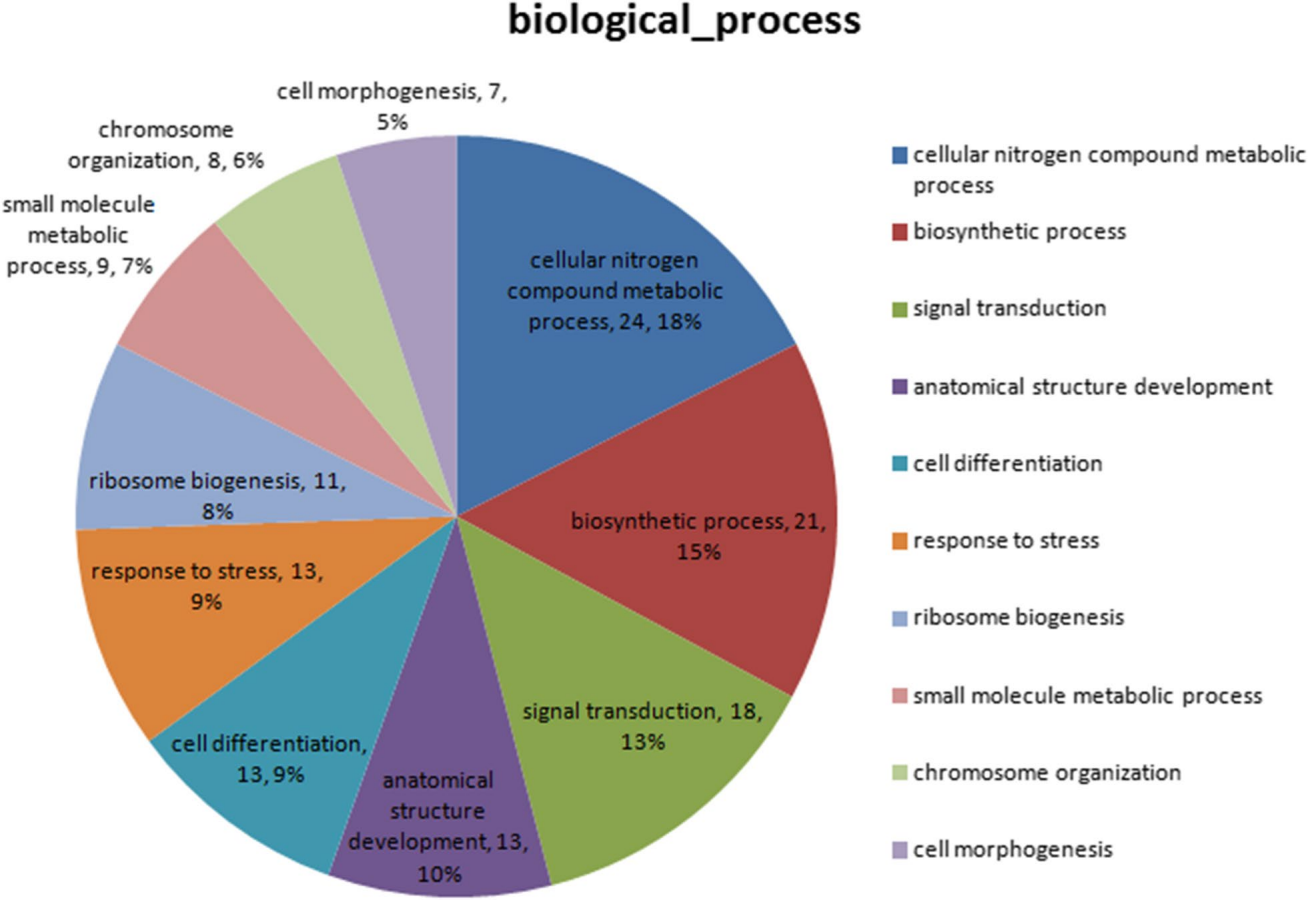
Functional annotation of wild-type ataxin-1-interacting proteins in HEK-293T cells. GO analysis was performed on 61 candidate wild-type ataxin-1-interacting proteins

It was essential to analyze the function of the candidate wild-type ataxin-1-interacting proteins. Sixty-one candidate proteins that interacted with wild-type ataxin-1 were identified and functionally annotated. The relative proportion of these proteins that were in candidate wild-type ataxin-1-interacting proteins in each separate functional group is shown as a series of pie charts (Fig. 2). The first group included cellular nitrogen compound metabolic processes, which are located on the right of the twelve o’clock position in the pie chart (for orientation). Biosynthetic processes, signal transduction, anatomical structure development, and cell differentiation were primary functional classifications also indicated in the pie charts.

**Fig. 3:**
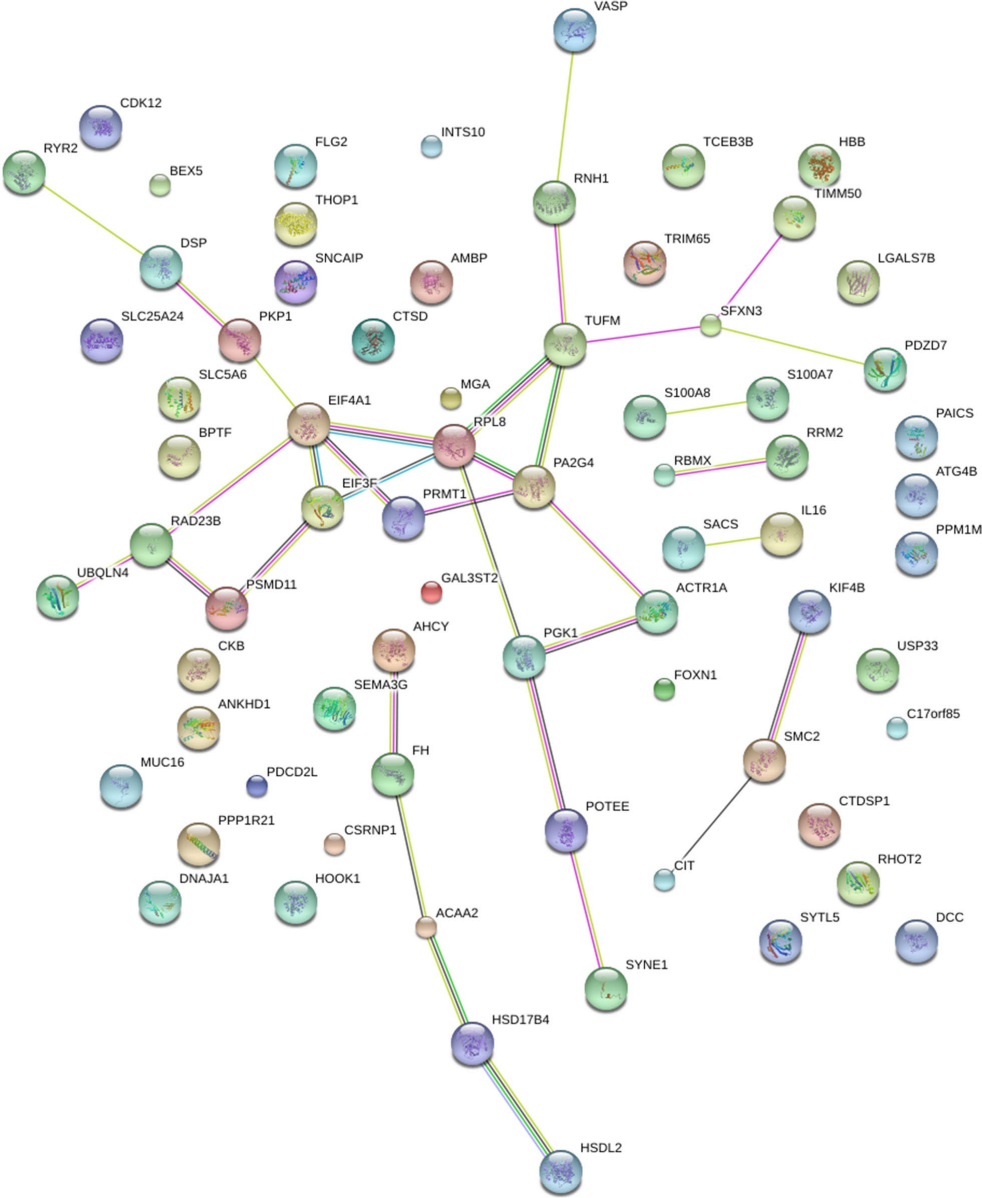
Protein–protein interaction network analysis of 61 candidate wild-type ataxin-1-interacting proteins in HEK-293T cells. Lines connecting the proteins suggest molecular relationships. The purples lines indicate experimental evidence; the yellow lines indicate text mining evidence; the green lines indicate gene neighborhood; the blue lines indicate gene co-occurrence database evidence; the black lines indicate the co-expression evidence

The candidate ataxin-1-interacting proteins were integrated based on information from the String database to construct a protein–protein interaction network (Fig. 3). The ribosomal protein, L8 (RPL8, line = 13), and eukaryotic translation initiation factor 4A1 (EIF4A1, line = 12) were the two most important hub nodes identified in the network. Thus, RPL8 and EIF4A1 exhibited the highest degrees of connectivity. Proliferation-associated protein 2G4 (PA2G4), mitochondrial Tu translation elongation factor (TUFM), and eukaryotic initiation factor 3 subunit F (EIF3F) were the second most important nodes in the network. Network analysis revealed that the candidate ataxin-1-interacting proteins were involved in RNA binding, DNA binding, enzyme binding, and cytoskeletal protein binding in HEK-293T cells.

### ChIP-seq identified ataxin-1 binding targets in an axon guidance pathway in HEK-293T cells

**Fig. 4:**
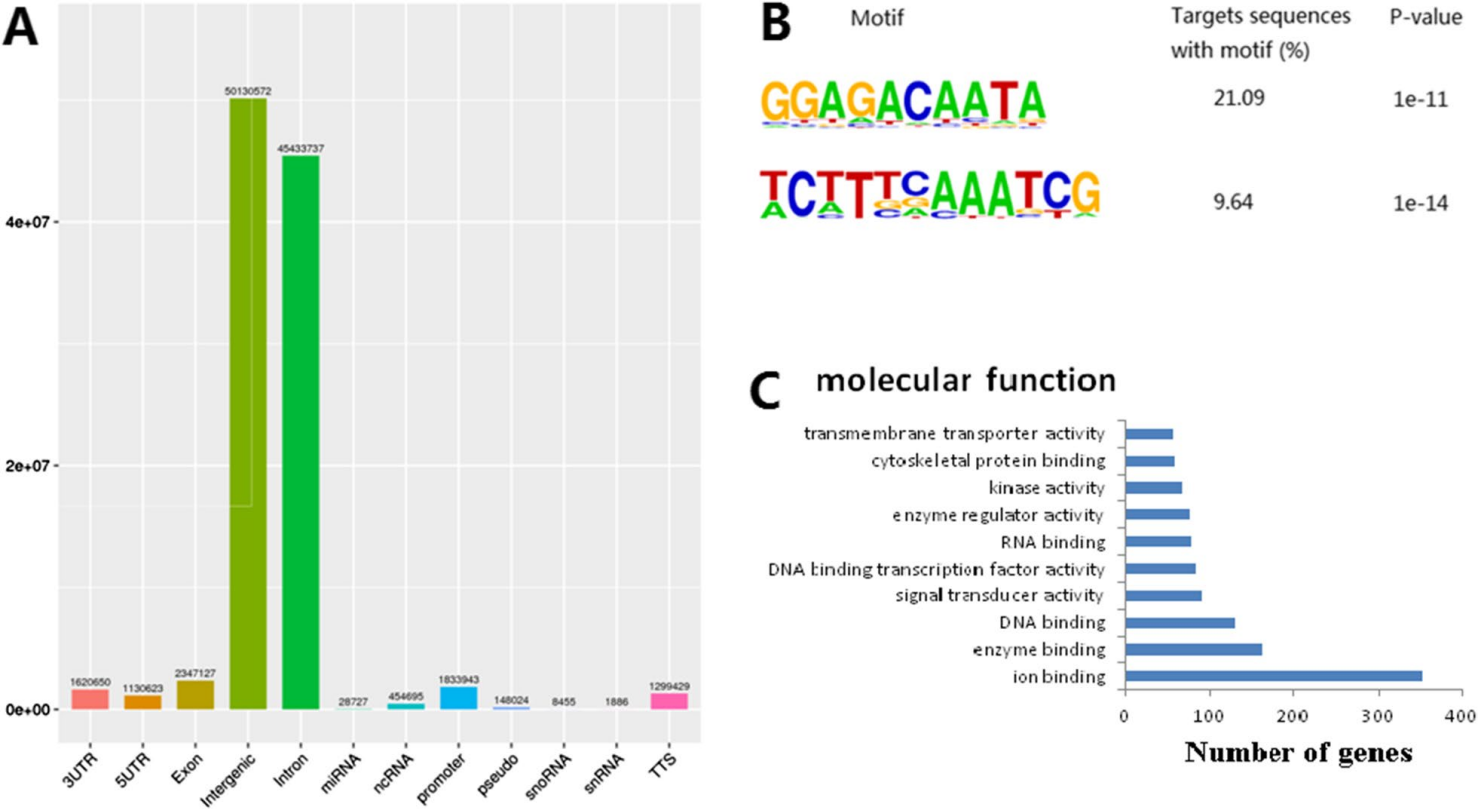
Genome-wide ataxin-1 binding profile and GO analysis in HEK-293T cells expressing wild-type ataxin-1. **A** The distribution of wild-type ataxin-1 binding sites in the genome. Peaks were distributed into intergenic, introns, exons, promoters, 3' UTRs, TTSs, 5' UTRs, and other functional elements. **B** The top two enriched short sequence motifs identified near ataxin-1 peaks throughout the genome. **C** GO analysis of the genes immunoprecipitated by ataxin-1 antibody in HEK-293T cells. The 1632 genes bound by ataxin-1 were subjected to GO analysis

Genomic DNA was immunoprecipitated with ataxin-1 antibody in HEK-293T cells. Genomic enrichment sites were sequenced using Illumina Hiseq2000. Analysis of the ChIP-seq data identified 95,327,876 short reads (https://www.ncbi.nlm.nih.gov/Traces/study/?acc=PRJNA688986) from wild-type ataxin-1 immunoprecipitated samples, and 112,809,382 short reads (https://www.ncbi.nlm.nih.gov/Traces/study/?acc=PRJNA688991) from mutant ataxin-1 immunoprecipitated samples. Among the short reads, 90,304,073 wild-type ataxin-1 ChIP-Seq reads and 106,709,192 mutant ataxin-1 ChIP-Seq reads were aligned to the human reference genome using the BOWTIE aligner. The peaks of the wild-type ataxin-1 binding sites were distributed into intergenic, introns, exons, promoters, 3' untranslated regions (3' UTRs), transcription termination sites (TTSs), 5' untranslated regions (5' UTRs), and other functional elements (Fig. 4A). The peaks of the mutant ataxin-1 binding sites were similar to wild-type ataxin-1 binding sites. There were 1632 putative target genes bound by wild-type ataxin-1, and only one putative target gene bound by mutant ataxin-1 using stringent statistical criteria in HEK-293T cells.

**Fig. 5:**
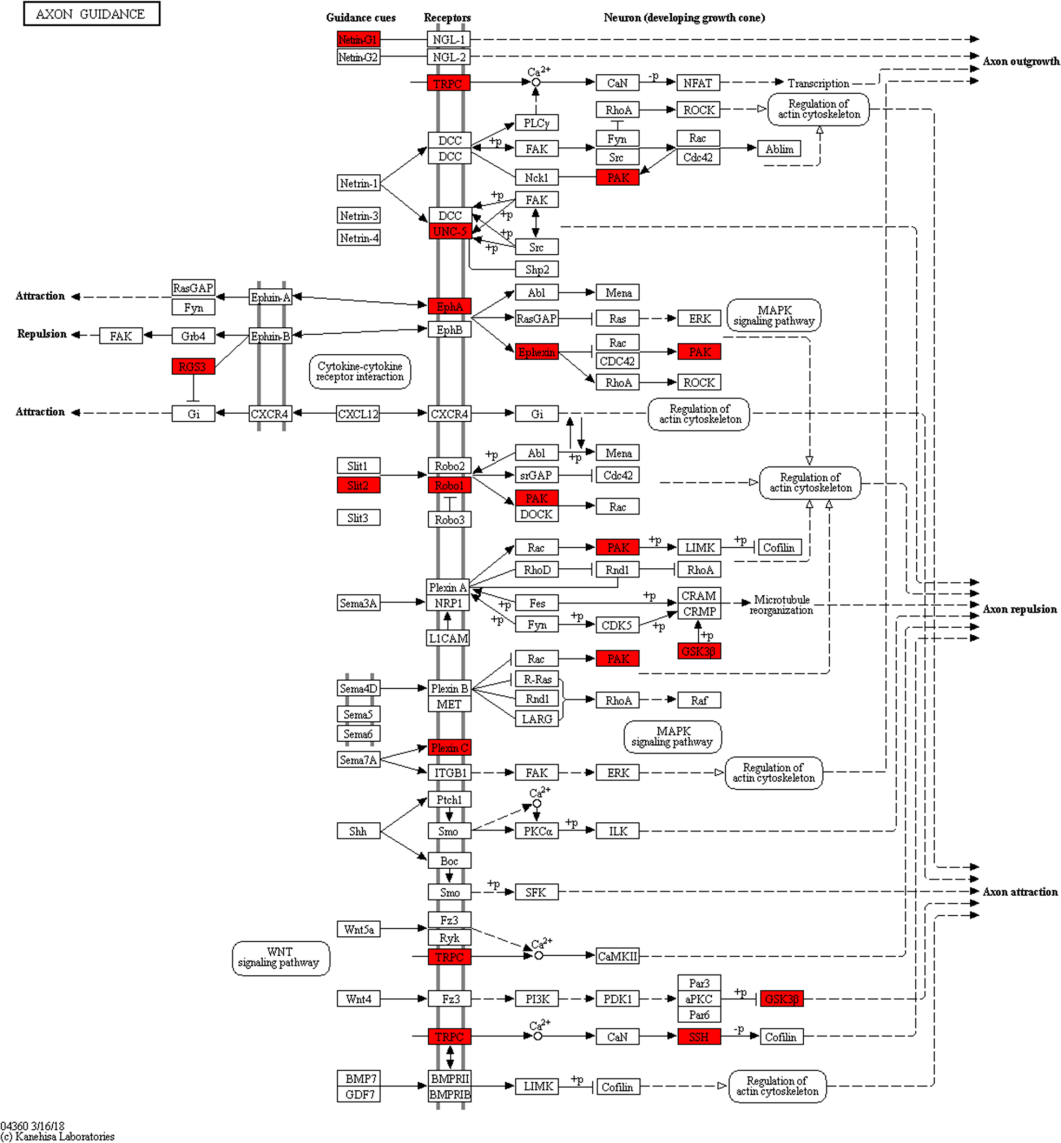
KEGG “axon guidance” pathway relevant to ataxin-1 target genes identified in HEK-293T cells by ChIP-seq. Entrez Gene IDs of 1632 ataxin-1 target genes were imported into the Core Analysis tool of the Ingenuity Pathway Analysis. It extracted the “axon guidance” pathway as the first rank significant pathway. Ataxin-1 target genes are highlighted by red

We then characterized the potential DNA binding motifs in the target sequences. The two most frequent motifs enriched in wild-type ataxin-1 binding targets contained the core GGAG (*p* = 10^−11^) and AAAT (*p* = 10^−14^), as shown in Fig. 4B. The 1573 protein-coding genes bound by wild-type ataxin-1 were subjected to GO analysis. The most highly enriched GO terms related to molecular functions were involved in ion binding (351 genes), enzyme binding (162 genes), DNA binding (130 genes), signal transduction activity (91 genes), and DNA binding transcription factor activity (84 genes) (Fig. 4C). The top 15 wild-type ataxin-1 binding genes with a peak score greater than six are listed in Table 2. Among the top 15 ataxin-1 binding genes, *SLC6A15*, *NTF3*, *KCNC3*, and *DNAJC6* were neuron-associated functional genes. The KEGG pathway enrichment analysis suggested that the network might be involved in axon guidance. Twelve identified genes were involved in the axon guidance pathway in HEK-293T cells (Fig. 5). Only one target gene was enriched using stringent statistical criteria in HEK-293T cells transfected with the plasmids pLVX-Puro-3xFlag-SBPtag2-Atx1-M that encoded the mutant ataxin-1. These observations suggested that the expansion of a CAG nucleotide repeat in the *ATXN1* gene might affect ataxin-1 binding to target genes.

**Table 2:** The top 15 ataxin-1 target genes with a peak score > 6 listed present in the GO pathway

Peak score	Gene name	Gene type	Annotation
10.4	SLC6A15	Protein-coding	Intergenic
8.84	H3F3AP4	Pseudo	Intergenic
8.73	NOTCH2NL	Protein-coding	Intron
8.51	ZNF733P	Pseudo	Intergenic
8.41	RGAG4	Protein-coding	5' UTR
7.97	IL15	Protein-coding	Intron
7.75	11-Mar	Protein-coding	Exon
7.42	GGT8P	Pseudo	Intergenic
7.1	PFN1P2	Pseudo	Intron
6.88	MIR1253	ncRNA	Intergenic
6.77	NTF3	Protein-coding	Intergenic
6.44	KCNC3	Protein-coding	Intron
6.44	DNAJC6	Protein-coding	Exon
6.33	NPAS4	Protein-coding	Intron
6.33	MAGEA11	Protein-coding	Intergenic

The peak score was calculated from Homer software (v4.6). Higher scores indicate more enrichment

### Ataxin-1 is an RNA-binding protein identified by RIP-sequencing

**Fig. 6:**
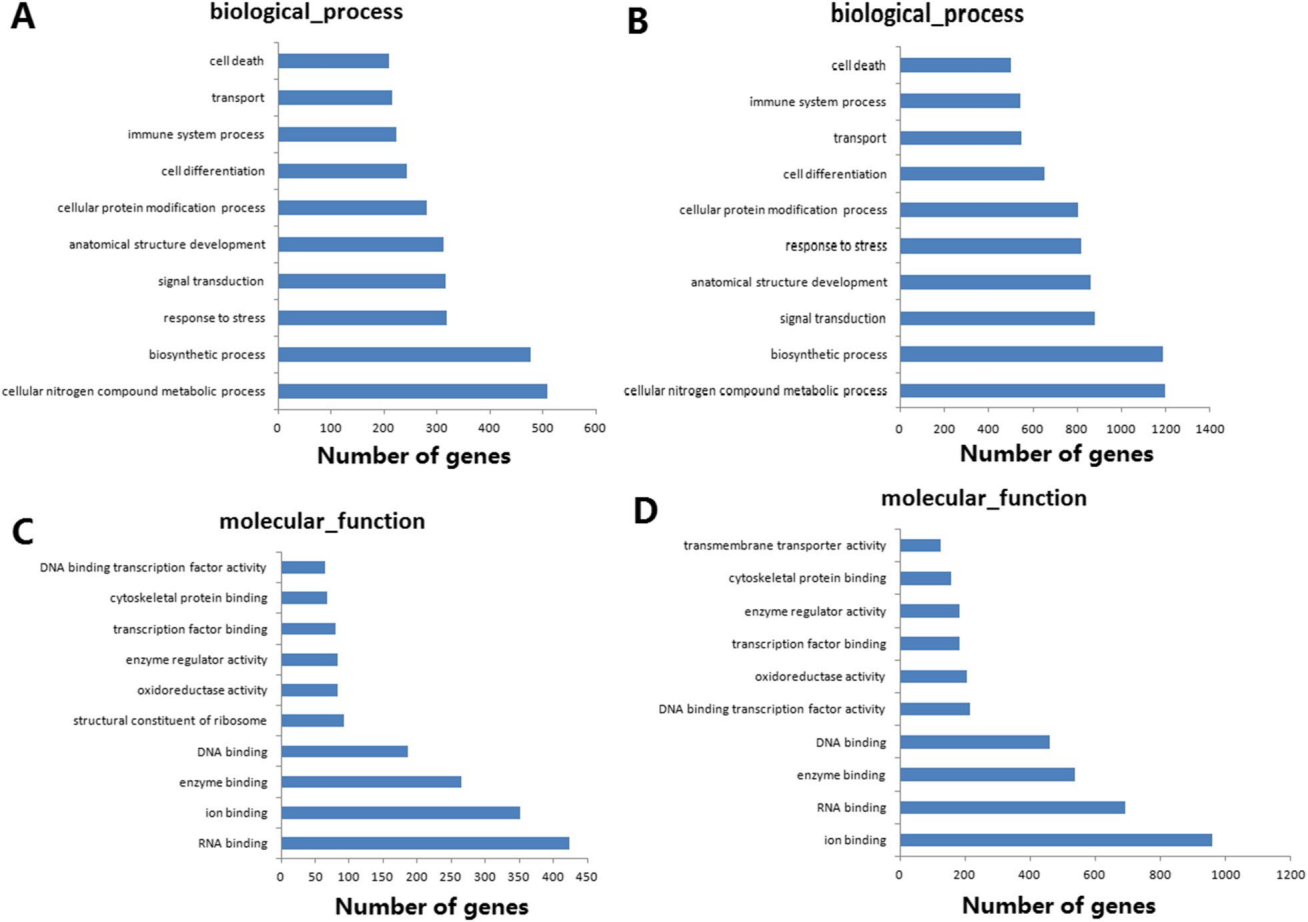
GO analysis for biological processes and molecular functions of wild-type ataxin-1-enriched RNAs and mutant ataxin-1-enriched RNAs. The length of each bar is proportional to the statistical significance of the enrichment. The number of ataxin-1 targets is displayed below the bar. **A** GO analysis for biological process of wild-type ataxin-1-enriched RNAs. **B** GO analysis for biological process of mutant ataxin-1-enriched RNAs. **C** GO analysis for molecular function of wild-type ataxin-1-enriched RNAs. **D** GO analysis for molecular function of mutant ataxin-1-enriched RNAs

To explore whether the expansion of a CAG nucleotide repeat affected the binding of ataxin-1 to target RNAs, RIP-sequencing was performed to identify endogenous RNA targets of wild-type and mutant ataxin-1 in HEK-293T cells. The efficiency of the immunoprecipitation of wild-type and mutant ataxin-1 was validated by Western blots using HEK-293T cells. The reads were processed using a standard bioinformatics protocol for RIP data (https://www.ncbi.nlm.nih.gov/Traces/study/?acc=PRJNA688985). The GO analysis demonstrated enrichment in biological processes linked to cellular nitrogen compound metabolic processing, biosynthetic processes, response to stress, signal transduction, and anatomical structure development in HEK-293T cells expressing wild-type ataxin-1 (Fig. 6A). The GO analysis also revealed enrichment in biological processes linked to similar processes in HEK-293T cells expressing mutant ataxin-1 (Fig. 6B). Molecular function analysis showed enrichment in wild-type- and mutant ataxin-1-bound transcripts for genes encoding proteins associated with RNA binding, ion binding, enzyme binding, and DNA binding (Fig. 6C and D). However, RNA binding was the most highly enriched molecular function in HEK-293T cells expressing wild-type ataxin-1 (Fig. 6C), and ion binding was the most highly enriched molecular function in HEK-293T cells expressing mutant ataxin-1 (Fig. 6D). Therefore, this study suggested that the CAG-repeat expansion in the *ATXN1* gene did not affect the RNA-binding activity of ataxin-1.

## Discussion

SCA1 is a fatal neurodegenerative disease induced by brain-region-specific cell death and dysfunction, and is characterized by impaired motor balance and coordination [9]. SCA1 is caused by a polyglutamine expansion in ataxin-1 which is involved in gene transcription. Transcriptional regulators including Capicua, Rbm17, SMRT, Gfi-1, and RORa/Tip-60, have been shown to interact with ataxin-1 [6, 7, 11–13]. Previous studies have explored how polyglutamine-expanded ataxin-1 affected the normal transcriptional signature in the cerebellum. These studies have found that various biological pathways including calcium signaling, glutamate signaling, and long-term depression are involved in the cerebellum at different stages [12–14]. Also, interacting protein partners can affect ataxin-1 stability and function [14]. The best-studied example is 14-3-3 protein, and its interaction with phosphorylated ataxin-1 in the cytoplasm can prevent ataxin-1 dephosphorylation and degradation and inhibit the ataxin-1 nuclear translocation required for its toxicity [15].

A better understanding of the pathogenesis of SCA1 requires a comprehensive assessment of the ataxin-1 interactome. Ataxin-1-interacting proteins were isolated using co-immunoprecipitation and screened by LC−MS/MS in HEK-293T cells expressing wild-type and mutant ataxin-1. GNAS, MCM2, and TMEM206 were identified as wild-type ataxin-1-interacting proteins using Western blots in HEK-293T cells. No mutant-ataxin-1-interacting proteins were pulled down in HEK-293T cells in our study. GNAS, MCM2, and TMEM206 were not reported as ataxin-1-interacting proteins in a previous study. Certainly, the microenvironment associated with HEK-293T cells might affect ataxin-1 post-translational modifications, ultimately affecting its molecular interactions. Currently, it is not clear whether ataxin-1 interacts with GNAS, MCM2, and TMEM206 in Purkinje cells since phosphorylation of S776 is associated with a stabilization of ataxin-1 [8]. Co-immunoprecipitation using a phospho-mimetic ataxin-1 or phospho-resistant ataxin-1 may be helpful to clarify the ataxin-1-interacting proteins in Purkinje cells.

Three new components of the ataxin-1 interactome required additional interrogation. GNAS mediates receptor-stimulated cAMP signaling which integrates different environmental cues with intracellular responses [16]. Mutations in the *GNAS* gene result in pseudohypoparathyroidism type 1a, pseudohypoparathyroidism type 1b, pseudopseudohypoparathyroidism, progressive osseous heteroplasia, polyostotic fibrous dysplasia of bone, McCune-Albright syndrome, Albright hereditary osteodystrophy, and several tumors, although the pathogenic mechanisms remain elusive [17]. A previous study reported that the presence of mutant GNAS is critical for a pancreatic tumor which is driven by protein kinase A (PKA)-mediated suppression of salt-inducible kinases [18]. So, we infer the *GNAS* mutation may affect PKA kinase activity, and these changes may happen in protein–protein interaction networks. Phosphorylation of ataxin-1 at the serine 776 residue plays an essential role in protein toxicity, and PKA is a critical kinase for ataxin-1-pS776 in cerebellar Purkinje cells [19]. Thus, our results suggested that ataxin-1-pS776, which is driven by the PKA pathway, might be mediated by GNAS-PKA interaction networks in neurons. The present study also revealed that RAC-PAK pathway is indeed a target gene of ataxin-1 in the KEGG axon guidance. Our study demonstrates the phosphorylation of ataxin-1 may involve in the pathogenesis of SCA1. These data provide further insight into how RAC-PAK pathway regulates ATXN1 levels in vitro and neurodegeneration in vivo. Together, these findings raise the possibility that GNAS may get involved in the pathogenesis of SCA1.

MCM2 is the highly conserved mini-chromosome maintenance protein involved in recruiting other DNA replication-related proteins and the formation of replication forks [20]. MCM2 forms a complex with MCM4, 6, and 7 and regulates the helicase activity of the complex. MCM2 is phosphorylated by protein kinases CDC2 and CDC7 [20]. A previous investigation revealed that MCM2 promotes cell proliferation, possibly through the regulation of HMGA1 phosphorylation [21]. A role for MCM2 in neurons has not been reported. Thus, the role of the MCM2/ataxin-1 interaction in neurons needs further investigation.

TMEM206 regulates the progression of colorectal cancer by promoting colorectal cancer cell proliferation and controlling colorectal cancer cell migration and invasion [22]. The TMEM206 target may be AKT, which is involved in regulating the biological behaviors of some cancers [22]. TMEM206 is a component of the proton-activated Cl^−^ channel that mediates Cl^−^ influx and is involved in acid-induced cell death [22]. A knockout of TMEM206 in neurons in mice attenuated brain damage after ischemic stroke [23]. We speculated that the pathological mechanism in SCA1 might be partially mediated by TMEM206, which was associated with the expansion of the CAG-repeat in the *ATXN1* gene, and led to chlorine influx-induced neuron death. TMEM206 is an unreported ataxin-1-interacting protein, and its molecular function in neurons should be explored in the future. In this study, the polyglutamine expansion in ataxin-1 led to its inability to interact with other partner proteins. This result suggested that the polyglutamine tract of ataxin-1 was essential to allow interactions with its protein partners.

Ataxin-1 functions as a regulator of transcription in the nucleus, and it regulates different transcription processes in concert with transcriptional modulators [24]. Transcriptional derangements precede the pathologic and behavioral features associated with SCA1 and mutant ataxin-1 causes alterations in gene expression in mouse models [24]. To identify target genes for ataxin-1 in wild-type and mutant conditions, we performed ChIP-seq in HEK-293T cells expressing wild-type and mutant ataxin-1. Our experiment identified a comprehensive set of wild-type ataxin-1 mRNA partners. The top two motifs contained the core, GGAG and AAAT, and were enriched in the ataxin-1-binding targets in HEK-293T cells expressing wild-type ataxin-1. The 1573 protein-coding genes were associated with wild-type ataxin-1 and identified using high-throughput sequencing. *SLC6A15*, *NTF3*, *KCNC3*, and *DNAJC6* were functional neuronal genes among the top 15 ataxin-1 binding genes identified using ChIP-seq.

The *SLC6A15* gene encodes a member of the solute carrier family 6 protein family, which plays an essential role in amino acid transport in neurons and might be associated with major depression [25]. SLC6A15 expression is specific to the brain and revealed a strong preference for branched-chain amino acids and methionine transport [25]. Amino acids play integral roles in the central nervous system as neurotransmitters, neuromodulators, and regulators of metabolism [26]. PKC activation can reduce the plasma membrane expression of the SLC6A15 protein [27]. Research presented in this study found that the *SLC6A15* gene is an interacting mRNA partner of wild-type ataxin-1. Our studies indicated that ataxin-1 might have a potential role in amino acid transport and ataxin-1 dysfunction that alters amino acid transport might contribute to SCA1 onset.

NTF3 is a protein member of the neurotrophin family that controls neuron survival and differentiation [28]. The NTF3 protein belongs to both brain-derived neurotrophic factor and nerve growth factor, which are involved with maintaining the adult nervous system and neuronal development in embryos [28]. NTF3-deficient mice displayed severe movement defects of the limbs [29]. Ataxin-1, a candidate binding protein for the *NTF3* gene, might be involved in the movement defect observed in NTF3-deficient mice through protein–DNA interactions.

The physiological function of KCNC3 in the cerebellum is well known [30]. Purkinje cells express KCNC3 in both their soma and dendrites, and KCNC3 plays a critical role in the Purkinje cell spikelet repolarization and the shaping of the complex spike [30]. Mutations in the *KCNC3* gene cause cerebellar neurodegeneration and impair auditory processing, termed spinocerebellar ataxia type 13 (SCA13) [31]. Our results determined that ataxin-1 binds the *KCNC3* gene, which suggests that the mutant ataxin-1 might contribute to the onset of SCA13 by regulating *KCNC3* gene transcription.

DNAJC6 is a brain-specific protein with 970-amino acids that is enriched in presynaptic termini; it belongs to the conserved DNAJ/HSP40 family of proteins, which regulate molecular chaperone activity by stimulating ATPase activity [32]. The DNAJC6 protein has three distinct domains including a conserved 70-amino acid domain at the N terminus that allows for its interaction with Hsc70, a cysteine-rich domain containing four motifs resembling a zinc finger domain, and a glycine/phenylalanine-rich region. In cells depleted of DNAJC6, vesicle trafficking is disrupted between the ER and Golgi, as well as throughout the Golgi [32]. A previous functional study demonstrated that ataxin-2 played a role in endocytic pathways associated with endosomal trafficking [33]. Our study suggested that ataxin-1 might affect vesicle trafficking mediated by DNAJC6.

Axons need to be correctly guided to their target during brain development [34]. Axon guidance allows the formation of intricate neural circuits that control the function of the brain [34]. Faulty disintegration and assembly of these circuits result in disorders of the nervous system. Some studies have demonstrated that axon guidance signaling pathways control gene expression through localized translation and transcription [34]. Among the 1573 protein-coding genes identified by the ataxin-1 by ChIP-seq, twelve were implicated in axon guidance. Axon guidance is mediated by a range of extracellular guidance contacts that include secreted factors and cell adhesion molecules [35]. The importance of axon guidance contacts and their receptors can be revealed based on links between mutations in genes that encode proteins associated with neurodegenerative diseases including Alzheimer’s disease, Parkinson’s disease, and amyotrophic lateral sclerosis [35]. The GO analysis identified ataxin-1 binding genes that were involved in axon guidance. Thus, we inferred that axon guidance disruption might be involved in the pathogenesis of SCA1. Our results also indicated that mutant ataxin-1 with the polyglutamine expansion nearly completely lost the ability to bind target genes. This result suggested that the normal polyglutamine tract of ataxin-1 was essential for protein–DNA interactions, and an abnormal expansion of polyglutamine led to SCA1.

RNA-binding proteins regulate RNA processing, including pre-mRNA splicing, mRNA transport, 3′end formation, translation, and degradation [36]. Ataxin-1 functions to regulate transcription and RNA processing in the nucleus [37]. In this study, mapping of RNA–protein interactions was performed using RIP-seq of the expression of wild-type and mutant ataxin-1 in HEK-293T cells. The GO analysis confirmed that the top two enriched biological processes were linked to cellular nitrogen compound metabolic processing and biosynthetic processes for wild-type and mutant ataxin-1. We also confirmed that the abnormal polyglutamine expansion did not affect on the ability of ataxin-1 to bind target RNAs. The GO analysis also identified the top two enriched molecular functions, which were linked to RNA binding and ion binding for wild-type and mutant ataxin-1, respectively. However, the most enriched molecular function was RNA binding for wild-type ataxin-1. On the other hand, the most enriched molecular function was ion binding for mutant ataxin-1. These data indicated that the polyglutamine expansion in ataxin-1 had little effect on the ability of ataxin-1 to bind target RNAs.

## Conclusions

In summary, using TAP tagging and co-immunoprecipitation-Western blot assays, we observed that ataxin-1 was bound GNAS, MCM2, and TMEM206 in HEK-293T cells. Two ataxin-1 binding targets containing the core, GGAG or AAAT, were identified in HEK-293T cells using ChIP-seq, and *SLC6A15*, *NTF3*, *KCNC3*, and *DNAJC6* were neuron-related genes among the top ataxin-1 binding genes. Therefore, an expanded polyglutamine tract in ataxin-1 might interfere with protein–protein or protein–DNA interactions but had little effect on protein–RNA interactions. This study suggested that the dysfunction of protein–protein or protein–DNA interactions is involved in the pathogenesis of SCA1.

## Data Availability

The datasets analyzed during the current study are available from the corresponding author on reasonable request.

## Abbreviations

SCA1: Spinocerebellar ataxia type 1
CIC: Transcriptional regulator Capicua
RBM17: RNA-binding motif protein 17
U2AF65: The splicing factor U2AF 65 kDa subunit
TAP: Tandem affinity purification
MCM2: Mini-chromosome maintenance complex component 2
GNAS: Guanine nucleotide-binding protein G subunit alpha isoforms short
TMEM206: Transmembrane protein 206
ChIP-seq: Chromatin immunoprecipitation high-throughput sequencing
RIP-seq: RNA immunoprecipitation high-throughput sequencing
KEGG: Kyoto Encyclopedia of Genes and Genomes
GO: Gene Ontology
PCR: Polymerase chain reaction
LC–MS/MS: Liquid chromatography-mass spectrometer/mass spectrometer
HPLC: High-performance liquid chromatography
AGC: Automatic gain control
RPL8: Ribosomal protein, L8
EIF4A1: Eukaryotic translation initiation factor 4A1
PA2G4: Proliferation-associated protein 2G4
TUFM: Tu translation elongation factor
EIF3F: Eukaryotic initiation factor 3 subunit F
3' UTRs: 3' Untranslated regions
TTSs: Transcription termination sites
5' UTRs: 5' Untranslated regions
PKA: Protein kinase A
SCA13: Spinocerebellar ataxia type 13
